# Nod-Like Receptors: Key Molecular Switches in the Conundrum of Cancer

**DOI:** 10.3389/fimmu.2014.00185

**Published:** 2014-04-23

**Authors:** Andrew Kent, J. Magarian Blander

**Affiliations:** ^1^Immunology Institute, Department of Medicine, Graduate School of Biological Sciences, New York, NY, USA; ^2^Tisch Cancer Institute, Icahn School of Medicine at Mount Sinai, New York, NY, USA

**Keywords:** nod-like receptors, cancer, immunoediting, immunosurveillance, innate immunity, transformation

## Abstract

It is believed the immune system can contribute to oncogenic transformation especially in settings of chronic inflammation, be activated during immunosurveillance to destroy early neoplastic cells before they undergo malignant outgrowth, and finally, can assist growth of established tumors by preventing clearance, remodeling surrounding tissue, and promoting metastatic events. These seemingly opposing roles of the immune system at the different stages of cancer development must all be mediated by innate signaling mechanisms that regulate the overall state of immune activation. Recently, the cytosolic nod-like receptor (NLR) pathway of innate immunity has gained a lot of attention in the tumor immunology field due to its known involvement in promoting inflammation and immunity, and conversely, in regulating tissue repair processes. In this review, we present all the current evidence for NLR involvement in the different stages of neoplasia to understand how a single molecular pathway can contribute to conflicting immunological interactions with cancer.

## Introduction

The pervading conception of the immune system today depicts it simply as the body’s means of warding off infection. In her *Anthropology of Immunology*, Martin eloquently describes “the body as nation state at war over its borders, containing internal surveillance systems (encompassed in the immune system) to monitor foreign intruders” (1). However, this “infection-centric” view does not consider profound facets of the immune system, now well established in the literature, and largely forgotten since the earliest immunologists predicted their existence. As early as the 1890s, Ilya Metchnikoff conceived of the theory of “physiological inflammation,” in which the immune system, especially phagocytic cells, were essential for maintaining homeostasis within all tissues of the body (2). He postulated that phagocytic cells uphold the balance between competing cell types and organs as they arise within a multicellular organism, establishing a unified “organismal identity” (2). This did not ignore the role of phagocytes in fighting infection, but suggested a “wide functional spectrum, of which host defense against pathogens was only one aspect” (2). Included were roles in regulating tissue development, clearance of damaged tissue, promotion of wound repair after any insult, be it infectious or sterile, and resolution of unwarranted inflammatory processes.

There is no better example of a question of organismal identity, of the need for a restoration of homeostasis, or of cell types or tissues in competition with one another, than that of cancer. Because they are initially derived from self-tissue, transformed cells pose a dilemma – to destroy or repair? It seems the immune system is responsible for answering this question, and is now known to be intimately involved in the oncogenic process from the very emergence of the first transformed cells through to malignant disease (3–5). Due to the nature of the predicament at hand, the immune system has been described to have conflicting roles depending on which stage of cancer progression is being studied (6). How the opposing immunological phenotypes in cancer are controlled is not well known, but nod-like receptors (NLRs) have been implicated in various stages of the disease process and have the required capacity to act as key regulators of physiological and pathological inflammation (7–9). NLRs are initiators of the inflammasome pathway, a cytosolic signaling apparatus that canonically activates caspase-1, and IL-1β and IL-18 thereafter (10). NLRs can respond to both pathogen- and danger-associated molecular patterns (PAMPs and DAMPs, respectively), and the pathway has been shown to have important roles in mounting immune responses to both microbial pathogens and damaged self, as well as regulating tissue repair after damage (11, 12). Here, we will review the evidence for NLR involvement in the initial emergence of neoplastic lesions, in the control and destruction of transformed cells during a phase of immunosurveillance, and finally the immune shift to supporting growth of established disease. We will argue that the conflicting roles of the immune system during oncogenesis can be reconciled within the framework of Metchnikoff’s theory of immune control of tissue homeostasis, and that NLRs and their downstream signaling elements serve as key molecular switches in this process.

## Emergence of Transformation

Schreiber and colleagues categorized immune interaction with cancer into three stages of immunoediting: elimination by immunosurveillance mechanisms; equilibrium, when cancer attains a latent balance between aberrant growth and destruction; and escape, when the tumor overcomes suppression as an edited malignancy (13). Although overlooked in the “Three E’s model” of immunoediting, the involvement of inflammatory processes in the initial emergence of cancer is well established within the literature. Chronic inflammation is a major risk factor for neoplasia in the clinic, working to both disrupt the microenvironment to favor neoplastic outgrowth, and contribute to genetic instability and altered turnover rates of stromal cells, promoting accelerated emergence of malignant clones (14). Many studies have now implicated the inflammasome pathway and the NLRs in this context, but with contrasting influences depending on the context and specifics under scrutiny.

A predominant model used to study NLR and inflammasome contributions to carcinogenesis is the AOM/DSS model (15). DSS causes damage to the colonic epithelium, while AOM causes G-to-A mutations in DNA of cells undergoing DNA replication. Deficiency in NLRP6, an NLR primarily expressed in colonic myofibroblasts, resulted in decreased repair of the intestinal epithelium following DSS treatment, but conversely, was associated with increased epithelial colonocyte proliferation and transcript expression of molecules involved in cell cycle progression (16). Another study showed prolonged colitis and epithelial destruction in *Nlrp6*^−/−^ mice after DSS treatment was related to alterations in commensal microbiota, and was phenocopied when mice were deficient in any of the NLRP6 inflammasome components ASC (a common adapter to many inflammasomes), and caspase-1 (17). The IL-18 cytokine, cleaved into its biologically active form by activated caspase-1, has emerged as a key cytokine downstream of inflammasome activation that enables epithelial repair after damage, but also prevents cancer progression through its induction of the tumor suppressors STAT1 and IFN-γ (18). When treated with AOM/DSS, the resulting increased epithelial proliferation and exacerbated inflammation in *Nlrp6*^−/−^ mice led to accelerated outgrowth of colonic cancer (16). In addition to NLRP6, loss of NLR family members NOD1, NOD2, NLRP3, NLRC4, and NLRP12 has resulted in similar exacerbated colitis and accelerated rates of cancer (19–24). Together, results from these gut studies suggest NLRs and their associated inflammasome components are essential for controlling wound repair responses and preventing transformative events and unwarranted epithelial proliferation early in potentially neoplastic settings (20). Much work needs to be done to clarify the mechanisms of NLR regulation in these processes, especially their connection to regulation of epithelial regrowth.

Paradoxically, over-expression of NLR pathway components also drives cancer rather than suppresses its emergence. As might be predicted from the above evidence, the derepression of caspase-1 that occurs in *Casp12*^−/−^ mice results in accelerated recovery from colitis after DSS. However, after AOM/DSS, these mice have accelerated rather than decreased colorectal cancer development, a pathology linked to increased levels of inflammatory cytokine gene expression including *Il1b* (25). In a model of HCV infection, IL-1β production downstream of NLRP3 by hepatic macrophages was linked to chronic hepatitis (26). Similarly, CCl_4_ treated *Nlrp3*^−/−^ and *Asc*^−/−^ mice exhibited reduced levels of liver fibrosis, and wild-type hepatic stellate cells treated with monosodium urate crystals upregulated the *Tgfb* and *Col1a* genes in an inflammasome-dependent manner (27). Thus in the liver, NLRs contribute to chronic inflammatory processes, both infectious and sterile, that result in the hepatitis and fibrosis commonly found prior to hepatocellular carcinoma.

IL-1β has many pleiotropic effects involved in inflammation, immunosuppression, cell proliferation and differentiation, tissue regeneration, tumor-promotion, and chemoresistance (28). In addition to its roles in hepatic carcinoma, the cytokine has been implicated in accelerating tumor development in mammary epithelial (29), gastric (30), and skin (31) cancer models, further establishing its role as an inflammatory instigator of oncogenesis. Drexler et al. were able to show both anti- and pro-tumorigenic effects of ASC in a single model of chemically induced skin carcinogenesis (31). ASC expression in infiltrating myeloid cells helped drive carcinogenesis, while ASC expression in keratinocytes suppressed epithelial cell proliferation and carcinogenesis (although in a caspase-1-independent manner). While the specific NLR implicated in these opposing roles of ASC was not identified, involvement of the inflammasome pathway was strongly implicated.

These studies all demonstrate opposing roles of the inflammasome in the early initiation of neoplastic disease. NLR activation can inhibit malignant transformation by controlling epithelial cell regeneration, but can also contribute to chronic inflammation that eventually results in carcinogenesis. The NLRs mediate a fine balance between inflammation and repair to maintain homeostasis in each tissue. If tipped in either direction, malignancy can result.

## Elimination of Transformed Cells

Once a transformed cell appears, it immediately presents a unique challenge to the immune system. Its uncontrolled proliferation threatens the evolutionarily defined healthy function of the tissue of its origin. Although derived from self, it no longer obeys the rules of organismal identity. From observations of homograft rejection, and increased cancer incidence in immunocompromised individuals, Lewis Thomas and Sir MacFarlane Burnett postulated the theory of immunosurveillance – the ability of the immune system to recognize and destroy abnormal self despite its ontogenic origins (32). Schreiber and others have built a strong case for the existence of adaptive immunosurveillance, and now evidence is emerging in spontaneous models of neoplasia (33–36).

Every adaptive response requires innate priming, thus innate immunity must be involved. Some studies have shown innate cell involvement (34, 37, 38), but thorough examinations of the molecular pathways that enable immune activation against tumor antigens are scarce. However, there are a few studies directly demonstrating NLRs can be involved in immunosurveillance. In an allograft model, Ghiringelli et al. show that chemotherapeutic killing of tumor cells causes a release of ATP that binds the P2RX7 purinergic receptor on dendritic cells (DCs), eventually leading to the activation of the NLRP3 inflammasome in these cells (37). By synergizing with HMGB1, released from dying tumor cells and signaling through toll-like receptor (TLR) 4, activated DC are licensed to prime an anti-tumor immune response in a caspase-1- and IL-1β-dependent manner. Another study found that extracts from an anti-tumorigenic mushroom functioned by activating the same P2RX7/NLRP3 pathway in macrophages, but did not draw a direct link to altered tumor kinetics (39). Although these conclusions derive from experimental models, anthracycline-treated breast cancer patients with mutations in the *P2rx7* gene were found to develop metastatic disease faster than those with normal *P2rx7* genes, suggesting the NLRP3-dependent pathway may be activated in humans with spontaneous disease (37). In addition to NLRP3, in 2012 we published on the ability of flagellin to synergistically activate TLR5 and the NLRC4 inflammasome, resulting in effective priming of CD4 and CD8 immunity against subcutaneously implanted allografts in mice (40). Besides priming of adaptive immunosurveillance, NLRs have been implicated in anti-tumor immunity through the link between IL-18 and increased NK cell activity against tumors (41–44). However, these latter findings were made in the presence of exogenous administration or expression of IL-18 above normal levels.

All these studies involve some artificial intervention that enhances NLR activity, but present a strong case for the ability of the pathway to influence immunosurveillance. It remains to be shown if the inflammasome pathway is involved in intrinsic immunosurveillance mechanisms, or is activated at this early stage of disease in any capacity. It is difficult to capture the elimination phase due to its transience and lack of overt disease phenotypes. Spontaneous models with a definable pre-malignant stage must be employed to further analyze which innate signaling pathways, and in which cell types, are naturally engaged to clear transformed cells before they cause disease. Selectively enhancing this engagement could greatly benefit therapeutic intervention. Additionally, these studies suggest a critical function of the inflammasome in priming adaptive immunity against transformed self-cells. It remains to be shown if this ability is mediated entirely through cytokine production, or if the inflammasome can influence T cell priming in a more direct manner. Conversely, it is possible there are strictly innate-mediated immunosurveillance or tumor-suppressing mechanisms engaged that help inhibit malignancy without priming T or NK cells (45). NLR involvement in these processes is unknown.

## Maintenance of Established Disease

Malignant disease is the result of failed immunosurveillance mechanisms. The editing process selects for clones of the rapidly dividing and mutating transformed cell that are progressively less immunostimulatory (13). Eventually, the developing tumor attains a phenotype that no longer incites immune destruction and can grow uncontrolled. Furthermore, established tumors are known to usurp immune mechanisms to not only prevent destruction, but facilitate growth (46). Tumors have been described as wounds that will not heal due to their self origin, the stress they undergo as they rapidly expand, and their elicitation of reparative and protective immune functions (47, 48).

In light of this analogy, it is not surprising to find NLRs activated in malignant disease, in this context attempting to repair the “wound” to restore homeostasis and protect it from further immune destruction. A host of evidence supports various roles for NLR-activated IL-1β in malignancy, notably in humanized models (49, 50). Okamoto et al. found that malignant human melanoma cells spontaneously activated their intrinsic NLRP3 inflammasome, resulting in caspase-1 cleavage and spontaneous secretion of IL-1β (51). This secreted IL-1β became increasingly autonomous with later stage disease, implicating it as an evolutionarily advantageous trait for the developing tumor. *In vitro*, the inflammasome pathway and IL-1β were shown to increase macrophage chemotaxis and angiogenesis, both features linked to worse prognosis in various cancers (52). Another study found that IL-1β and caspase-1-deficient mice were much less susceptible to melanoma liver metastases by an injected allograft, improving their overall survival (53). *In vitro*, secreted factors from the melanoma cell line induced IL-18-dependent upregulation of VCAM-1 on hepatic sinusoidal endothelial cells, as well as IL-1β secretion. In opposition to the results in the previous section, endogenous IL-18 from melanoma cells was also found to inhibit NK cell-mediated killing of melanoma cells by upregulating Fas ligand expression (54). Additionally, IL-18 was found to enhance immunosuppression of NK cells by inducing upregulation of the inhibitory molecule PD-1 (55).

Nod-like receptors are also implicated in the ability of myeloid-derived suppressor cells (MDSCs) to inhibit anti-tumor immunosurveillance. Related to the gut studies in the first section, IL-1β over-expression in the stomach was shown to induce inflammation and cancer (30). This was associated with an increase in MDSC numbers homing to the stomach in an IL-1R and NF-κB-dependent fashion. In a model of DC-based vaccination against melanoma, van Deventer et al. demonstrated that *Nlrp3*^−/−^ mice had improved outcomes due to decreased numbers of MDSCs homing to the tumor site (56). However, they did not observe a change in MDSC function, such as the ability to suppress T cell responses. Finally, chemotherapy was found to trigger cathepsin B release within MDSCs, triggering NLRP3 within the same cells (57). The resultant IL-1β production induced IL-17 secretion by CD4 T cells. Allograft tumor growth was slower in *Il17a*^−/−^, *Il1r1*^−/−^, *Nlrp3*^−/−^, and *Casp1*^−/−^ mice after chemotherapy treatment, demonstrating all elements in this pathway play a part in tumor protection although the exact mechanism is unclear.

This evidence clearly implicates the NLRs and inflammasome pathway in tumor-promotion and defense. They directly facilitate tumor cell growth and metastasis, and help prevent any anti-tumor immune responses. It is curious to speculate how accurate the analogy of tumor to “unhealing wounds” is with regards to NLR involvement. Are NLRs engaged in the same way by malignant disease as they are by damaged tissues prior to malignant transformation, in both cases inducing repair and protective properties? Fitting with the tumor editing hypothesis, any pro-inflammatory DAMPs or other signals resulting from initial transformation that would trigger tumor clearance have in theory been selected away, leaving only those characteristic of damaged self in need of repair. Inflammasome involvement in such diverse functions as tissue repair, immune suppression, and inflammation warrants a search for more inflammasome-activated targets besides IL-1β and IL-18 that could fine-tune downstream effector mechanisms. Are these two cytokines alone able to control such diverse effects, or are they working in collaboration with many other pathways, the overall milieu defining the result? Concerted efforts to consolidate information across tumor models and treatments, being mindful of cell-type specificity, will help clarify these points.

## Conclusion

**Figure 1:**
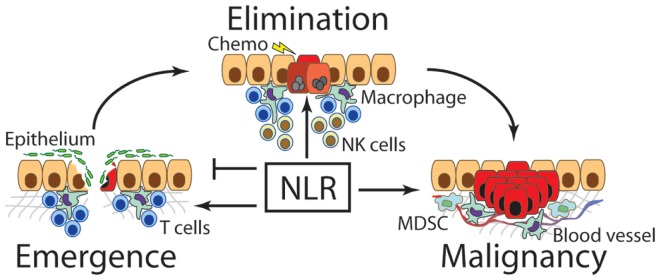
**Nod-like receptors contribute to the emergence, elimination, and maintenance of cancer**. The first transformed cells emerge under some form of oncogenic stimulus such as chronic inflammation. At this stage, NLRs have been found to regulate repair of damaged tissue, especially the rate or re-epithelialization, as well as the degree of inflammation to most appropriately clear invading pathogens. Over or under-expression of NLRs and their downstream signaling molecules can lead to increased incidence of cancer emergence. After a transformed cell emerges, NLRs are thought to contribute to immunosurveillance and destruction of newly transformed cells, especially in combination with chemotherapeutics or other immunological interventions. Finally, once a malignant cancer clone escapes suppressive mechanisms, NLRs support the tumor by facilitating neovascularization, aiding metastasis, and promoting MDSCs and other immunosuppressive functions.

We have now seen how NLRs switch roles in every stage of cancer progression (Figure 1). In each, the NLRs can be conceptualized as attempting to restore homeostasis. First, in situations where damage to self has occurred, the NLRs contribute both to fighting off infection and repairing the damaged epithelial layers. The latter implicates an ability of the NLR pathway to regulate growth of surrounding tissues, with a strong link to IL-18. These processes require perfect coordination to maintain equilibrium in the tissue. The fact that too much or too little NLR signaling in this type of setting can result in neoplasia betrays how essential this pathway is to maintaining balance and organismal integrity. Second, when the very idea of self is challenged by oncogenic mutations, again NLR signaling is observed. Presumably here in early pre-neoplastic situations, NLR activation functions as an innate defense against localized transformation events. When clinical pathology is observed, these endogenous protective functions of the NLR have failed. Therapeutic enhancement of this activation has been shown to be beneficial in mouse models, especially in concert with activation of other inflammatory pathways such as TLRs. Thus, development of therapies that employ NLRs could have great impact in the clinic, especially if used very early in neoplasia. Finally, after tumors become established and are immunologically indistinguishable from other self-tissues, NLR activation reverts to helping protect and maintain this neo-self, establishing a new, pathological state of homeostasis. Malignant disease is extremely hard to treat in part because of this unique pseudo-self phenotype and consequent immunoprotective state, reiterating the need for early intervention for successful treatment. Metchnikoff’s prescient description of physiological inflammation is thus embodied within the recently discovered NLR pathway. Theories from this founding father of immunology can still help us conceptualize the perplexing and, in the case of NLRs and cancer, diametrically opposed functions of the immune system.

## Conflict of Interest Statement

The authors declare that the research was conducted in the absence of any commercial or financial relationships that could be construed as a potential conflict of interest.
